# Comparison of wall thickening and ejection fraction by cardiovascular magnetic resonance and echocardiography in acute myocardial infarction

**DOI:** 10.1186/1532-429X-11-22

**Published:** 2009-07-09

**Authors:** Martha Nowosielski, Michael Schocke, Agnes Mayr, Kathrin Pedarnig, Gert Klug, Almut Köhler, Thomas Bartel, Silvana Müller, Thomas Trieb, Otmar Pachinger, Bernhard Metzler

**Affiliations:** 1 Clinical Division of Cardiology, Innsbruck Medical University, Innsbruck, Austria; 2 Department of Radiology I, Innsbruck Medical University, Innsbruck, Austria

## Abstract

**Objectives:**

The purpose of this study was to compare cardiovascular magnetic resonance (CMR) and echocardiography (echo) in patients treated with primary percutaneous coronary intervention (PCI) for acute myocardial infarction (AMI) with emphasis on the analysis of left ventricular function and left ventricular wall motion characteristics.

**Methods:**

We performed CMR and echo in 52 patients with first AMI shortly after primary angioplasty and four months thereafter. CMR included cine-MR and T1-weighted first-pass and late-gadolinium enhancement (LGE) sequences. Global ejection fraction (EF_CMR_, %) and regional left ventricular function (systolic wall thickening %, [SWT]) were determined from cine-MR images. In echo the global left ventricular function (EF_echo_, %) and regional wall motion abnormalities were determined. A segment in echo was scored as "infarcted" if it was visually > 50% hypokinetic.

**Results:**

EF_echo _revealed a poor significant agreement with EF_CMR _at baseline (r: 0.326; p < 0.01) but higher correlation at follow-up (r: 0.479; p < 0.001). The number of infarcted segments in echocardiography correlated best with the number of segments which showed systolic wall thickening < 30% (r: 0.498; p < 0.001) at baseline and (r: 0.474; p < 0.001) at follow-up. Improvement of EF was detected in both CMR and echocardiography increasing from 44.2 ± 11.6% to 49.2 ± 11% (p < 0.001) by CMR and from 51.2 ± 8.1% to 54.5 ± 8.3% (p < 0.001) by echocardiography.

**Conclusion:**

Wall motion and EF by CMR and echocardiography correlate poorly in the acute stage of myocardial infarction. Correlation improves after four months. Systolic wall thickening by CMR < 30% indicates an infarcted segment with influence on the left ventricular function.

## Background

Ischemic heart disease has become the leading cause of death from cardiovascular disease worldwide [1]. Therefore the diagnosis of myocardial infarction, early treatment through primary angioplasty and the evaluation of regional cardiac function by means of cardiac imaging are of increasing importance. The most important clinical application of regional functional analysis is the assessment of reversibly injured but still viable myocardium in ischemic heart disease [2,3]. Cardiovascular magnetic resonance (CMR) detects myocardial stunning from wall motion abnormalities. Gadolinium based contrast agents are used to assess myocardial perfusion, to identify nonviable myocardium and scarring, and to estimate the extent of infarction [4,5] ensuring minimal interobserver and intraobserver variability [6]. The exact measurement of infarct size may provide valuable information on ventricular remodelling, arrhythmic potential, and prognosis and is a commonly used surrogate endpoint for the evaluation of new therapies for acute myocardial infarction (AMI) [7]. Several studies reported an "overestimation" of infarct size based on contractile function in acute myocardial infarction [8], mostly due to viable tissue surrounding the infarct [9-12]. Mahrholdt et al [13] described an "inverse tethering", an underestimation of infarct size, in chronic myocardial infarction due to contraction of neighbouring segments. This inevitably leads to difficult interpretation of data concerning early and late infarct size as well as left ventricular wall characteristics [14]. However, the clinical assessment of regional function – in particular, of myocardial viability and stress-induced ischemia – is based on the subjective assessment of wall motion [15]. Although the quantitative measurement of wall thickening provides a more precise parameter of regional function than visual estimation of wall motion by echocardiography (echo) [8], the latter is the most commonly used tool to assess cardiac morphology and function in daily clinical practice [16].

Few studies have directly compared echo with CMR in patients with AMI [16,17] – a comparison that forms a basis for the interpretation of functional results using different imaging modalities [18]. In the following study we compared these two cardiac imaging modalities by using the AHA 17-segment model in patients with first AMI shortly after primary percutaneous coronary intervention (PCI) and four months thereafter. Our three main focal points are on the evaluation of left ventricular function, the evaluation of left ventricular wall motion characteristics and its improvement within a follow-up period of 4 months.

## Methods

### Patient population

Fifty-two patients (44 men and 8 women) admitted to the coronary care unit (CCU) at the Innsbruck University Hospital, presenting with first AMI defined by elevated cardiac enzymes and an ECG with ST-segment elevation ≥ 2 mm in 2 continuous electrocardiographic leads, were eligible for enrolment. Patients were included if they had a) no previous history of myocardial infarction b) a "Thrombolysis in Myocardial Infarction" (TIMI) flow of less than 3 prior to p-PCI) c) a TIMI flow of 3 after percutaneous revascularisation in the affected coronary artery and had no contraindications to CMR.

The study protocol was approved by the hospital ethics committee, and informed consent was obtained from each patient.

CMR and echocardiographic investigations were performed in all patients shortly after (3 ± 2 days) p-PCI and approximately four months after reperfusion (131 ± 45 days).

Mean delay, defined as "onset of pain-to-balloon" time, was 4.6 ± 5 hours, ranging from 0.5 to 24 hours. All patients were treated with p-PCI and six received thrombolysis prior to mechanical revascularisation. Seven patients had to be excluded because of a delay lasting longer than 24 hours. Three patients had to be excluded because of claustrophobia during CMR, and in two CMR scans, no gadolinium was given. Those patients were excluded from the study prior to statistical analysis. Table 1 summarises the clinical characteristics of the patient population.

**Table 1:** Clinical parameters

**Study variable**	**Values, mean ± SE**
Age (yrs)	54 ± 12
Patients (number)	52
Men (number)	44
Body mass index (kg/m^2^)	25.1 ± 2.8

Days between AMI to baseline scan	

2D	3.4 ± 1.7
CMR	2.8 ± 1.6

Days between baseline and follow-up imaging	

2D	131 ± 45
CMR	132 ± 45

Delay, (h)	4.6 ± 5
cTnT, ug/l (max)	6.43 ± 4.26
CK, U/l (max)	2172 ± 1468

Infarct localisation	

posterior wall	31
anterior wall	18
lateral	3

Pre-hospital thrombolysis	6

2D findings	

EF (%), baseline	51.2 ± 8.1
EF (%), follow-up	54.5 ± 8.3
Segments evaluated	832
"infarcted" segments, baseline	2.1 ± 1.6
"infarcted" segments, follow-up	1 ± 1

CMR findings	

EF (%), baseline	44.2 ± 11.6
EF (%), follow-up	49.2 ± 11.1
Infarct mass (g), baseline	19.5 ± 12.2
Infarct mass (g), follow-up	15.8 ± 11.1
Segments showing LGE, baseline	7.4 ± 3.1
Segments showing LGE, follow-up	7.3 ± 3

Clinical characteristics of study patients; AMI: acute myocardial infarction, 2D: 2 dimensional echocardiography, CMR: cardiac magnetic resonance, Delay: door-to-balloon-time, cTnT: cardiac Troponin T, CK: creatine kinase, EF: ejection fraction, LGE: late enhancement. Data is presented as mean ± standard error (SE)

### CMR protocol

Using a 1.5 Tesla MR scanner (Magnetom Avanto, Siemens, Erlangen, Germany) providing total imaging matrix, cine-MR images in short- and long-axis were acquired during breath-hold, with retrospective ECG-triggered trueFISP (Fast Imaging with Steady-State Precession) bright-blood sequences with generalized autocalibrating partial parallel acquisition (GRAPPA, acceleration factor: 2) reconstruction. The patients were positioned into the spine array coil and covered by an 8-channel array coil, resulting in a total of 16 array elements for signal collection. Since it is known that signal acquisition with the help of multiple receiver coil systems can be deranged by the non-uniformity of coil sensitivities [19], we employed the prescan normalization function for intensity inhomogeneity correction.

The short-axis sequence contained 11 slices and had a repetition time (TR) of 46.8 ms, an echo time (TE) of 1.1 ms, a slice thickness (SL) of 8 mm, a field of view (FoV) of 350 × 263 mm, a matrix of 320 × 260, and a flip angle (FA) of 71°. The long-axis sequence contained 3 slices with a TR of 54.5 ms, a TE of 1.28 ms, a SL of 6 mm, a FoV of 360 × 293 mm, a matrix of 320 × 260, and a FA of 71°.

Each patient underwent dynamic bolus tracking with the help of an ECG-triggered single-shot trueFISP and a Gadolinium bolus of 0.1 mmol/kg body weight (Gd-DTPA, Magnevist, Schering, Berlin). The contrast bolus was injected into the cubital vein with a flow rate of 5 mL/s by using a commercially available MR injector (Spectris, Medrad, Pittsburgh, PA).

The dynamic trueFISP had a FoV of 380 × 265 mm, a SL of 8 mm, a matrix of 128 × 96, a TR of 172 ms, a TE of 0.96 ms and a GRAPPA iPat factor of 2.

After a minimum of 10 min. and an additional contrast bolus (another 0.1 mmol/kg body weight) was applied. Late Gadolinium Enhancement (LGE) CMR was performed on the same scanner by using a phase-sensitive inversion recovery single-shot balanced steady-state free precession sequence with consecutive slices perpendicular to the short axis with a TR of 590 ms, a TE of 1.2 ms, a SL of 8 mm, an inter-slice gap of 2 mm, a FoV of 400 × 363 mm, a matrix of 256 × 232, a FA of 45°, and a GRAPPA iPat factor of 2.

### Post-processing of CMR

Planimetry of LGE images was performed off-line using a commercially available software tool (J-Vision Vs. 3.3.16, TIANI). A threshold of +5 SD above the signal intensity of normal myocardium in the opposite non-infarcted myocardial segment was set to define the extent of LGE. Infarct volume [cm^3^] was calculated by multiplying the LGE area with slice thickness including the inter-slice gap.

Volumetric evaluation was performed using standard software (ARGUS, Siemens Erlangen, Germany). Contouring of left ventricular endo- and epicardial borders was performed semi-automatically. Left ventricular myocardial mass was calculated by multiplying the wall volume and the specific density of cardiac muscle (1.05 g/cm^3^).

Myocardial systolic segmental wall thickening (SWT) analysis was performed for each slice on the basis of the same endo- and epicardial contours. For each segment, end-diastolic and end-systolic wall thickness (EDWT, ESWT [mm]) as well as end-diastolic to end-systolic wall thickening (SWT, [mm] or [% of EDWT]) were assessed and calculated. SWT was summed and then averaged for each segment from the short axis slices involved by this segment. All segments were divided into groups according to their SWT, resulting in 5 groups with SWT of 20, 30, 40, 50 and 60%. Subsequently, these groups were correlated to infarcted segments in echocardiography, segments showing late enhancement, infarct mass in grams and EF_CMR _and EF_echo_. See Table 2.

**Table 2:** Correlation of SWT scores with CMR findings and infarcted segments in echo

**Baseline**					**Follow-up**			
**SWT**	**Number of infarcted segments** **in echo**	**EF** **Echo**	**EF** **CMR**	**Infarct mass in grams**	**Number of infarcted segments** **in echo**	**EF** **Echo**	**EF** **CMR**	**Infarct mass in grams**
**< 20%**	0.446	0.434	0.714	0.604	0.380	0.360	0.505	0.440
**< 30%**	0.498	0.449	0.744	0.591	0.474	0.382	0.561	0.545
**< 40%**	0.406	0.377	0.742	0.525	0.488	0.431	0.584	0.544
**< 50%**	0.316	0.364	0.762	0.475	0.533	0.448	0.597	0.513
**< 60%**	n.s.	0.386	0.737	0.369	0.555	0.470	0.633	0.500

Table 2 shows the correlation of different SWT (systolic wall thickening) scores, markers of regional wall motion abnormality, with the number of infarcted segments in echo (assessed visually, > 50% hypokinetic), late enhancement in CMR and EF in echo and CMR, in all values (p < 0.001). The best correlation of infarcted segments in echo and SWT in CMR is found at a SWT less then 30%. SWT < 30% correlates also highly significant with EF_CMR _and EF_echo_. Through this correlation we were able to describe a cut-off value of less than 30% SWT to define an infarcted segment in CMR with influence on the left ventricular function. Spearman test was used for linear correlations of the selected variables. n.s = not significant.

### Echocardiography

For transthoracic echocardiography a Sequoia 256 ultrasound unit (Acuson-Siemens Inc., Mountain View, California) was employed. Measurements of end-diastolic volume (EDV), end-systolic volume (ESV) and ejection fraction (EF, %) were done using standard software. The apical 5-chamber and 3-chamber views were used to calculate end-diastolic and end-systolic volumes according to the apical biplane summation-of-disks algorithm according to the guidelines by the American Society of Echocardiography using an average of two or three beats [20].

In the visual assessment of regional wall motion abnormalities, a segment was scored as infarcted if it was visually > 50% hypokinetic. The decision to use 50% as a cut-off value to define an infarcted segment in echocardiography was made by the authors out of practical reasons to achieve a higher accuracy of this visually based method. The visual assessment of wall motion abnormalities as well as the assessment of the EF were done by one highly experienced echocardiographer.

### Segmental model

All images (echocardiography and CMR) were analysed using the American Heart Association/American College of Cardiology (AHA/ACC) recommended 17-segment model [21] to provide adequate sampling of the left ventricle and coronary distribution without exceeding the resolution limits of the imaging modalities. Segment 17, the apex, was not analysed due to poor image quality of this part of the heart. In CMR two to three short axis images were assigned to either basal, mid or apical segments visually according to anatomic landmarks (papillary muscles) and 4-chamber view. In echocardiography standard cut planes were used to assess wall motion abnormalities and a moving grid representing the 17-segment model was placed in a certain position assigning segment 2 and 3 to the septum.

### Statistics

Analysis was performed with SPSS 16.0 for Windows (SPSS Inc., Chicago, IL). Normality was tested by Kolmogorov-Smirnov test. Pearson (if normally distributed (ND) and Spearman test (if not ND) were used for calculation of linear correlations for selected variables. With regard to the correlation of segments, paired Wilcoxon rank test was used to determine statistical significance of segmental improvement between baseline and follow-up. ANOVA with Bonferroni post-hoc testing (if normally distributed, ND) and Kruskal-Wallis test (K-W, if not ND) were used to determine differences between groups. A probability value of p < 0.05 was considered significant. Data is presented as mean ± standard error (SE) if not stated otherwise. Sensitivity and specificity were assessed by cross tabulation.

## Results

### Correlation of ejection fraction assessed by echo and CMR

**Figure 1:**
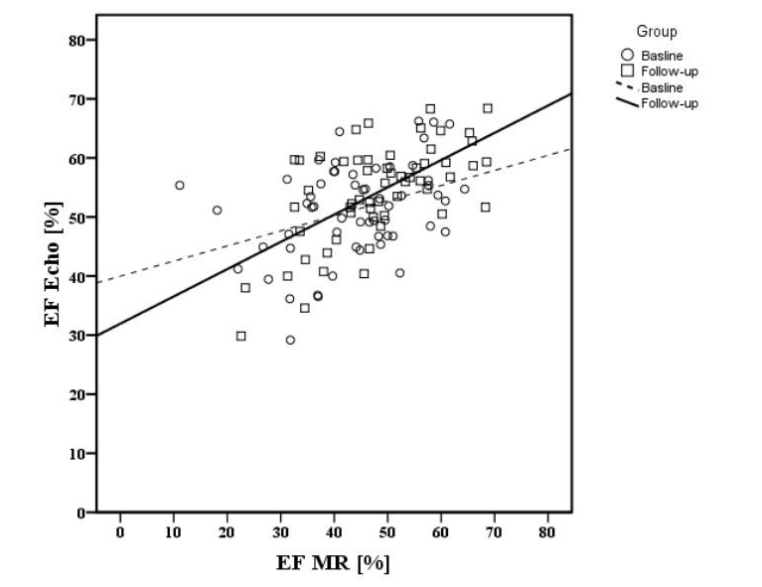
**Correlation of EF_echo _and EF_CMR _at baseline and at 4-month follow-up**. Figure 1 shows the correlation of EF (ejection fraction) between echo and CMR at baseline (dashed line, r: 0.326; p < 0.01) and at follow-up (continuous line, r: 0.479; p < 0.001). At follow-up echocardiography correlates better with CMR than at baseline.

EF in echocardiography (EF_echo_) correlated poorly with EF in magnetic resonance imaging (EF_CMR_) at baseline (r: 0.326; p < 0.01) but better at follow-up (r: 0.479; p < 0.001). (Figure 1). Comparison of EF_echo _(baseline: 51.2 ± 8.1%, follow-up: 54.5 ± 8.3%; p < 0.01) with EF_CMR _(baseline: 44.2 ± 11.6%, follow-up: 49.2 ± 11%) revealed that echo measured statistically significantly higher global EF than cine-CMR at baseline and at follow-up (p < 0.001).

### Wall motion abnormalities assessed visually by echo and semi-automatically by CMR

A comparison of wall motion abnormalities assessed by the two different imaging techniques revealed the following results. Systolic wall thickening in CMR, semi-automatically assessed, correlates in all values highly significant with the visually assessed wall motion abnormalities in echo. In echo a segment was scored as infarcted if it was > 50% hypokinetic. The best correlation between SWT in CMR and infarcted segments in echo could be detected at a SWT value less than 30% (r: 0.498; p < 0.001) Table 2 shows the individual values for the analysed segments.

### Correlation of SWT scores

SWT < 30% in CMR showed a highly significant correlation (p < 0.001) with EF_echo _and EF_CMR _(r: 0.449 and r: 0.744, respectively) and the infarct mass in grams (r: 0.591) at baseline and at follow-up (r: 0.382, r: 0.561 and r: 0.545, respectively.) Table 2.

A cross tabulation of segments showing LGE with different SWT scores revealed the following sensitivities for baseline: SWT < 20% (sensitivity 20.9%), SWT < 30% (33.9%), SWT < 40% (45.2%), SWT < 50% (56.6%), SWT < 60% (63.8%) and at follow-up: SWT < 20% (sensitivity 17.4%), SWT < 30% (27.4%), SWT < 40% (37.5%), SWT < 50% (49.3%), SWT < 60% (58.3%). See Table 3.

**Table 3:** Cross tabulation of various SWT scores with segments showing LGE in CMR

**Baseline**				**Follow-up**			
		**LGE negative**	**LGE positive**			**LGE negative**	**LGE positive**
**SWT < 20%**	negative	382	306	**SWT < 20%**	negative	381	313
			
	positive	63	81		positive	72	66
			
	sensitivity	20.9			sensitivity	17.4	
	specificity	85.8			specificity	84.1	

**SWT < 30%**	negative	342	256	**SWT < 30%**	negative	361	275
			
	positive	103	131		positive	92	104
			
	sensitivity	33.9			sensitivity	27.4	
	specificity	76.9			specificity	79.7	

**SWT < 40%**	negative	304	212	**SWT < 40%**	negative	324	237
			
	positive	141	175		positive	129	142
			
	sensitivity	45.2			sensitivity	37.5	
	specificity	68.3			specificity	71.5	

**SWT < 50%**	negative	252	168	**SWT < 50%**	negative	283	192
			
	positive	193	219		positive	170	187
			
	sensitivity	56.6			sensitivity	49.3	
	specificity	56.6			specificity	62.5	

**SWT < 60%**	negative	206	140	**SWT < 60%**	negative	239	158
			
	positive	239	247		positive	214	221
			
	sensitivity	63.8			sensitivity	58.3	
	specificity	46.3			specificity	52.8	

Table 3 compares each SWT (systolic wall thickening) score at baseline and follow-up through cross tabulation with the segments in CMR showing LGE (late enhancement). Sensitivity and specificity were calculated through cross tabulation.

### Transmurality and SWT

Mean SWT for non affected segments at baseline was 63,6% ± 20.3%. 448 segments showed no transmurality (group 1), 59 had a transmurality less than 50% (group 2) and 325 showed a transmurality over 50% (group 3). In order to detect differences between the transmural extent of the infarct and change of SWT, we made three groups according to their transmurality. In the acute state of myocardial infarction we detected a highly significant difference in SWT between the first group and the third group. Within the groups there could not be detected any difference regarding SWT and transmurality and there was no detectable difference between group 1 and group 2. Table 4

**Table 4:** Transmurality and SWT

**Transmurality**	**Number of segments**	**Mean SWT ± SE**	**Statistical difference to group 2 (< 50%)**	**Statistical difference to group 3 (> 50%)**
**group 1: 0**	448	63.6% ± 20.4%	n.s.	p < 0.001
**group 2: < 50%**	59	50.44% ± 48.7%	x	n.s.
**group 3: > 50%**	325	50.08% ± 20.0%	n.s.	x

In order to detect differences between the transmural extent of the infarct and changes of SWT (systolic wall thickening), we made three groups according to their transmurality (first had no transmurality, second had less than 50%, and the third had more than 50% transmurality). In the acute state of myocardial infarction we detected a highly significant difference in SWT between the first group with no scar and group 3 (p < 0.001). Within the groups there could not be detected any difference regarding SWT and transmurality. There could not be detected any difference between group 1 and group 2. n.s. = not significant, SE = standard error. ANOVA with Bonferroni post-hoc testing was used for statistical analysis.

### Improvement of left ventricular wall characteristics four months post AMI

**Figure 2:**
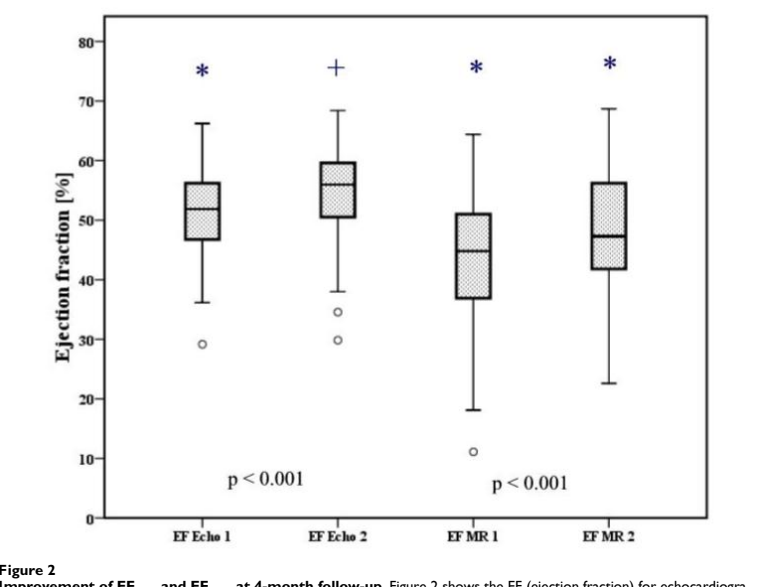
**Improvement of EF_echo _and EF_CMR _at 4-month follow-up**. Figure 2 shows the EF (ejection fraction) for echocardiography (51.2 ± 8.1%) and CMR (44.2 ± 11.6%, at baseline (EF echo 1, EF CMR 1) and follow-up (EF echo 2, EF CMR 2) (54.5 ± 8.3%, and 49.2 ± 11%, respectively) and visualises the statistically highly significant (p < 0.001) improvement at 4-month follow-up. (* p < 0.001, + p < 0.01). Data is presented as mean ± standard error and median.

Of the 832 evaluated segments (52 patients × 16 segments), improvement of the following parameters was highly significant (p < 0.001) after a four-month period. The total number of infarcted segments in echo decreased from 111 to 57. At baseline the mean EF_echo _was 51.2 ± 8.1% and 54.5 ± 8.3% at follow-up (p < 0.001). The mean EF_CMR _increased from 44.2 ± 11.6% to 49.2 ± 11% (Figure 2). The infarct mass in grams decreased from 19.5 ± 12.2 grams at baseline to 15.8 ± 11.1 grams at follow-up.

## Discussion

### Correlation of ejection fraction

In this study left ventricular function was measured both by echocardiography and CMR in the same patient population shortly after primary PCI in the setting of AMI and at four months follow-up.

A comparison of the two imaging modalities regarding ejection fraction revealed a poor correlation both at baseline and follow-up, with the agreement being better at follow-up (Figure 1). The difference between acute and follow-up correlations might be due to two conditions. In acute infarction, contractile function may be reduced due to myocardial stunning and accessorily by non-reperfused infarction due to ongoing ischemia despite adequate mechanical revascularisation as hypothesised by Marholdt et al. [13]. Since stunned myocardium – the result of an ischemic insult leading to contractile dysfunction [22] – resolves on a time scale of days to weeks [13], it can be expected to have no influence on contractile function at our 4-month follow-up investigation. Ongoing ischemia could be excluded as well, as we only enrolled patients with post-procedural TIMI-flow 3, i.e., those with successful revascularisation.

Furthermore, acute changes occurring in left ventricular loading conditions due to emergency medication or intravenous fluid administration are absent in the chronic scenario, which might also explain the differences in the EF readings obtained by both imaging modalities between acute and chronic conditions.

While interpreting the higher correlation in the chronic state of myocardial infarction it should be mentioned that 31 of the 52 patients presenting to the CCU with acute coronary syndrome had a posterior wall infarction. Since in biplane echocardiographic EF analyses contractility of the posterior wall may not fully account for EF, a slight overestimation in the calculation of the EF may result and might explain the higher echocardiographic EF values. This presumption is reinforced by a subanalysis showing a statistically highly significant difference (p < 0.001) between EF_echo _(54.1% at baseline and 55% at follow-up) and EF_CMR _(45.3% at baseline and 49.3% at follow up) in patients with posterior wall infarction. This difference was not detected in anterior wall or lateral infarction (no significant difference). CMR, with its capability for volumetric evaluation, is able to view the whole left ventricle and detect wall motion abnormalities even in regions inaccessible to echo and it is less likely operator dependent than biplane echo.

Ventricular and contractile function improvement has been reported to occur as a result of successful reperfusion [23-27]. Such improvement might be the reason for our observation that echo was a more accurate diagnostic instrument at follow-up (Figure 2) because of the previously mentioned omittance of the posterior wall in echo. After contractile improvement of the posterior wall this part of the heart might make a greater contribution to the EF, and therefore the correlation with CMR is more accurate.

Reichek et al. [28] presented preliminary results and determined LVEF in 28 patients after AMI by volumetric CMR and biplane echo, revealing a correlation of r: 0.43, which is similar to our results (baseline correlation r: 0.339; p < 0.001). Jenkins et al. [17] compared 2D, 3D and CMR in patients with healed myocardial infarction and found a high correlation between EF_echo _and EF_CMR _at 1 year follow-up of r: 0.70; p < 0.01. Thus, our findings on the higher grade of accuracy of echo under conditions of chronic myocardial infarction are in agreement with those reported in literature.

Another point that has to be taken into account is that echo revealed statistically significant higher EF values at baseline and at follow-up. This would imply an underestimation of infarct size in both settings, acute and chronic. This is an important fact that may influence clinical acting, as the EF is the most common consulted value to define left ventricular function, and has been proven to be an important indicator of prognosis after acute myocardial infarction [29-31].

### Systolic wall thickening in CMR

Animal studies have found that, independent of wall motion or infarct age, the size and the shape of regions showing late enhancement coincide with regions of myocardial necrosis and irreversible injury; regions, however, with a lack of LGE are viable [32,33]. Further studies showed that late enhancement detected within 24 hours after primary PCI in the infarct zone is an independent predictor of impaired left ventricular systolic thickening and remodelling [34,35].

In Table 3. we assessed a segment showing late enhancement as the gold standard to define an infarcted segment in CMR and compared it to different SWT scores. Through cross tabulation we could detect a relatively low, but increasing sensitivity the higher we set the SWT cut-off value. This shows that an infarcted segment, demonstrated through late enhancement, is always associated with a slight wall motion abnormality and therefore has a certain influence on the contractile function of the left ventricle. The crux is that not every wall motion abnormality leads to impaired left ventricular function and furthermore to a clinical deterioration. A SWT cut-off value had to be found in CMR that characterises a severely injured myocardial segment shortly after p-PCI ("infarcted" segment) with wide influence on the clinical outcome and bearing on the global ventricular function.

No prior study has defined the exact degree of reduction in SWT in CMR needed to be interpreted as "infarcted". Therefore, we attempted to find this degree of SWT reduction by correlating different SWT scores with the number of affected segments in echo, the infarct mass in grams and the ejection fraction (measured both with echo and CMR). The best correlation of infarcted segments in echo and SWT in CMR was found at a SWT less then 30%. SWT less than 30% also correlated also highly significant with EF_CMR _and EF_echo_. Through this correlation we were able to describe a cut-off value in CMR of less than 30% SWT to define an infarcted segment which has a bearing on the clinical outcome. Table 2. Furthermore we detected that the average SWT for healthy segments was 63.6%, so our cut-off value of 30% represents a 50% restriction of a normal SWT. This cut-off value has a specificity of 76.9% and a sensitivity of 33.9% and for us this value has a higher priority than the cut-off value analysed through ROC curves, which would be a SWT of 60% (with a sensitivity of 63.8% and a specificity of 46.3%).

### Transmurality and SWT in CMR

Choi et al. [27] reported that in patients with acute myocardial infarction, the transmural extent of infarction defined by CMR predicts improvement in contractile function. We found a highly significant difference in SWT between segments with no late enhancement and segments with a transmurality of more than 50%. Interestingly we could not detect any changes of SWT within the two groups with transmurality and between the first group with no transmurality and the second group with less than 50% transmurality. This might be due to the fact that contractile function in acute myocardial infarction is reduced because of myocardial stunning. Table 4.

### Limitations

Otterstad et al. reported that the coefficient of variation of EF_echo _is 15%, with 12% of this ascribable to test-retest variability [36]. In order to keep this inaccuracy as low as possible, all echocardiographic measurements in our study, baseline and follow-up, were done by one experienced observer.

Secondly, because of relatively short delays – 19 patients had a delay of less than 3 hours, 24 were within a range of 3 to 6 hours, 7 patients were within a range of 6 to 12 hours and 2 patients had a delay longer than 12 hours – the infarcted mass could be kept relatively small, as previously reported by our research group [14]. Myocardium showing only slight impairment or in some cases even no impairment (16 patients had an EF > 55%), may have been difficult to detect by echo. The correlation between echo and CMR may have been even more significant in more severely injured myocardium.

**In summary**, the results of our study show that CMR might be the more accurate method for assessing left ventricular function in the acute phase of myocardial infarction, whereas in the chronic state, also echocardiography can be a valuable and accurate instrument. Furthermore, systolic wall motion thickening in CMR at less than 30% can be used as a cut-off value to define an infarcted segment with influence on the left ventricular function.

## Competing interests

The authors declare that they have no competing interests.

## Authors' contributions

MN analysed and interpreted the provided data and designed and wrote the article. MS participated in the design of the study and performed the statistical analysis. AM carried out the volumetric evaluation on cine-MR images using ARGUS software. KP carried out the post-processing of CMR and performed the planimetry of LGE images. GK has made contributions to conception of the data and participated in the statistical analysis. AK carried out the echocardiographic measurements. TB revised the manuscript critically for intellectual content. SM revised the manuscript and completed the method section on echocardiography. TT participated in the design of the study. OP has given final approval to carry out the study. BM conceived of the study, coordinated and helped to draft the manuscript. All authors read and approved the final manuscript
